# Dysregulated serum lipid profile and its correlation to disease activity in young female adults diagnosed with systemic lupus erythematosus: a cross-sectional study

**DOI:** 10.1186/s12944-020-01232-8

**Published:** 2020-03-14

**Authors:** Bo Zhou, Yulong Xia, Jianqing She

**Affiliations:** 1 Respiratory And Critical Care Medicine, the First Affiliated Hospital of Xi’an Jiaotong University, Xi’an, 710048 People’s Republic of China; 2 Cardiovascular Department, the First Affiliated Hospital of Peking University, Beijing, 100005 People’s Republic of China; 3 Cardiology Department, the First Affiliated Hospital of Xi’an Jiaotong University, Xi’an, 710048 People’s Republic of China; 4 Key Laboratory of Environment and Genes Related to Diseases, Ministry of Education, Xi’an, 710048 People’s Republic of China

**Keywords:** Dyslipidemia, Lipid profile, Low-density lipoprotein, SLE disease activity index, Systemic lupus erythematosus

## Abstract

**Background:**

Recent studies showed that dyslipidemia could be a critical factor in the progression of cardiovascular disease in systemic lupus erythematosus (SLE). The aim of the present study was to describe the relationship between serum lipid profile and SLE disease activity in young female adults with SLE.

**Methods:**

Seventy-one female subjects diagnosed with SLE aged 20~30 years were enrolled. Serum lipid profile including TC, TG, HDL-C, LDL-C, VLDL-C, Apo A, Apo B, and Apo E were evaluated between control and young female SLE patients. Univariate correlation analyses were performed to explore the correlation between serum lipid levels and SLE disease activity.

**Results:**

Our results showed that TG and VLDL-C levels were significantly increased in young female SLE as compared to control, with TC, HDL-C, LDL-C, Apo A, and Apo B significantly reduced. Meanwhile, univariate correlation analyses showed negative correlations between SLE disease activity index and HDL-C, LDL-C, Apo A, and Apo B; with positive correlations between SLE disease activity index and TG and VLDL-C.

**Conclusion:**

Serum lipid profile was significantly dysregulated in young female SLE patients. Moreover, SLE disease activity was correlated to the serum lipid levels, supporting the notion that the young patients with SLE might also have a higher risk of cardiovascular disease.

## Introduction

Systemic lupus erythematosus (SLE) is a chronic inflammatory autoimmune disease, which is characterized by the deposition of the immune complex in multiple organs [1]. Nowadays, it has been proved that SLE is associated with increased risk of cardiovascular disease [2], which correlates to elevated mortality in the SLE patients [3]. Although chronic activation of the immune system in the patients with SLE could partially result in the development of atherosclerosis [4], the exact mechanisms underlying the progression of cardiovascular disease due to SLE itself were not fully understood.

Recent clinical studys showed that the patients with SLE had twofold higher risk of cardiovascular disease as compared to control, and SLE patients with lupus nephritis display significantly increased risk of myocardial infarction and cardiovascular disease mortality than SLE patients without lupus nephritis [5, 6]. Additionally, SLE disease-related parameters could be taken into consideration when calculating cardiovascular disease risks, and most of the patients with SLE were diagnosed with dyslipidemia, suggesting that dyslipidemia could be a critical factor in the progression of cardiovascular disease in SLE [7–10]. The patients with SLE exhibited increased total cholesterol (TC), triglycerides (TG) and very low-density lipoprotein-cholesterol (VLDL-C), and decreased high-density lipoprotein-cholesterol (HDL-C) and apolipoprotein A (Apo A) compared with control [11]. Also, it has been shown that VLDL-C and triglycerides were increased, and HDL-C and LDL-C levels were decreased in the patients with active SLE, by comparation with inactive SLE [12]. Thus, recent discoveries have further strengthened the critical role of SLE-related immune dysregulation and metabolic disturbances in accelerating cardiovascular disease. However, few studies have investigated the correlation between serum lipid profile and SLE disease activity.

As we known, traditional risk factors such as renal impairment and glucocorticoid treatment are correlated to dyslipidemia in the patients with SLE [13], but few studies have focused on the effect of disease itself and its activity in SLE population. In the present study, we evaluated lipid profile in young female adults newly diagnosed with SLE and the relationship between dyslipidemia and disease activity of SLE, indicating that disease activity of SLE itself could be an important modulator of dyslipidemia.

## Materials and methods

### Subjects

This study is a single centre, cross-sectional study. Seventy-one young female adults (20~30 years old) newly diagnosed with active SLE admitted to the First Affiliated Hospital of Xi’an Jiaotong University from 2013 January to 2017 December were enrolled in the present study. Once the patients were admitted to the hospital, demographic characteristics were collected; laboratory measurements including dsDNA, TC, TG, HDL-C, LDL-C, VLDL-C, Apo A, Apo B and Apo E were done by the diagnostic department and were forwarded to the hospital electronic system. Written informed consent was obtained from all subjects upon enrollment according to the Declaration of Helsinki, and was approved by the ethics committee, Xi’an Jiaotong University. All subjects met American College of Rheumatology (ACR) criteria for SLE. SLE activity was measured via the SLE Disease Activity Index 2000(SLEDAI-2 K) as a predictor for mortality and as a measure of global disease activity in the SLE [14–16]. The subjects were excluded if they: were taking or had previously taken any drugs known to influence lipid metabolism or the endocrine system in the past 6 months, such as lipid lowering agents, antihypertensives, estrogen, progesterone and anticonvulsants; received glucocorticoids or chloroquine or other immunosuppressive drugs; had diabetes mellitus, cardiovascular disease, liver and thyroid disease; had renal impairment or opportunistic infections; were with abnormal body mass index (BMI); had a history of smoking and alcohol use; were pregnant. A control group consisting of 71 healthy female adults between the ages of 20 and 30 years old was used to compare the results obtained, which were healthy and did not have any clinical condition.

### Laboratory measurements

The following laboratory assays were performed in the clinical laboratory department: TC and TG were detected using detection kit from FUJIFILM^(^™^)^ via HMMPS method; HDL-C, LDL-C and VLDL-C were detected using detection kit via direct measurement method from FUJIFILM^(^™^)^; Apo A, Apo B and Apo E was measured using detection kit from SEKISUI^(^™^)^ by turbidimetric inhibition immuno assay. All laboratory assays were performed in duplicate and the results were averaged.

### Statistical analysis

Data are shown as means ± standard error of mean (SEM) unless stated otherwise. Simple t test was used to compare continuous variables which are in normal distribution. Mann-Whitney U test was used to compare continuous variables which do not conform to the normal distribution. Data distribution normality was verified. Pearson′s correlation analysis was used to examine univariate correlation. Univariate correlation analyses were used to assess the correlations between SLEDAI and other parameters. Statistical analyses were performed using SPSS 20.0 software. Differences were considered significant when the *P*-value was less than 0.05.

## Results

### Clinical characteristics in the young female SLE and the control

As shown in Table 1, 71 subjects with SLE were evaluated ranging from 20 to 30 years old with normal BMI and normal serum creatinine levels. Age, BMI and serum creatinine levels did not significantly differ between the subjects with SLE and the control. Meanwhile, the subjects with SLE exhibited reduced C3 and C4 levels, and increased ESR, CRP and serum dsDNA antibody compared to the control. Additionally, SLE activity was evaluated by SLEDAI, and SLEDAI was 12.87 ± 2.81 in the subjects with SLE, suggesting active SLE in the cohort at the time of diagnosis.

**Table 1:** Clinical characteristics of the subjects with SLE and the control

Parameter	SLE (*n* = 71)	Control (*n* = 71)	Reference	*P*
Age (years)	27.6 ± 4.2	26.3 ± 3.9	20~30	0.24
Weight (kg)	48.2 ± 6.24	50.15 ± 4.87	/	0.14
Height (cm)	152 ± 8.63	151 ± 9.25	/	0.18
BMI (kg/m^2^)	19.8 ± 2.0	20.2 ± 1.9	20~25	0.21
Creatinine (umol/L)	68.3 ± 6.2	65.9 ± 5.8	35~71	0.09
ESR (mm/h)	40.34 ± 4.57*	3.28 ± 0.36	0~15	< 0.01
CRP (mg/L)	35.32 ± 3.29*	2.56 ± 0.19	0~10	< 0.01
SLEDAI (units)	12.87 ± 2.81*	0	0	< 0.01
C3 (g/L)	0.51 ± 0.08*	1.46 ± 0.05	0.80~1.85	0.03
C4 (g/L)	0.06 ± 0.03*	0.31 ± 0.04	0.10~0.40	0.02
dsDNA antibody (IU/mL)	98.54 ± 9.23*	1.85 ± 0.10	0~7	< 0.01

*BMI* Body mass index, *C3* Complement 3, *C4* Complement 4, *CRP* C-reactive protein, *ESR* Erythrocyte sedimentation rate, *SLEDAI* SLE disease activity index. Data are mean ± SEM. Statistically significant difference between the SLE subjects and the control (*P < 0.05)

### Lipid profile in the SLE subjects and the control

Next, the effects of SLE on lipid metabolism was assessed by lipid profile in the SLE subjects and the control. As show in Table 2, TG and VLDL levels were significantly increased in the subjects with SLE as compared to the control. Likewise, HDL-C and Apo A were markedly decreased in the SLE subjects by comparation with the control. Interestingly, the SLE subjects showed lower TC, LDL-C and apolipoprotein B(Apo B) levels. No significant difference was found in apolipoprotein E(Apo E) levels between these groups.

**Table 2:** Lipid profile in the SLE subjects and the control

Parameter	SLE (n = 71)	Control (*n* = 71)	Reference	*P*
Total cholesterol (TC, mmol/L)	3.10 ± 0.08*	4.27 ± 0.10	3.10~5.69	0.02
Triglyceride (TG, mmol/L)	2.22 ± 0.06*	0.82 ± 0.03	0.40~1.60	< 0.01
HDL-C (mmol/L)	0.83 ± 0.04*	1.56 ± 0.05	0.83~1.96	0.02
LDL-C (mmol/L)	1.68 ± 0.05*	2.53 ± 0.04	2.07~3.10	< 0.01
VLDL-C (mmol/L)	0.78 ± 0.03*	0.28 ± 0.02	0.08~0.41	< 0.01
Apolipoprotein A (Apo A, g/L)	0.88 ± 0.03*	1.32 ± 0.04	1.00~2.00	< 0.01
Apolipoprotein B (Apo B, g/L)	0.63 ± 0.02*	0.92 ± 0.04	0.60~1.10	0.03
Apolipoprotein E (Apo E, mg/L)	38.57 ± 1.29	36.62 ± 1.14	27.00~45.00	0.08

*HDL-C* High-density lipoprotein-cholesterol, *LDL-C* Low-density lipoprotein-cholesterol, *VLDL-C* Very low-density lipoprotein-cholesterol, and Data are mean ± SEM. Statistically significant difference between the SLE subjects and the control (*P < 0.05)

### Clinical correlation between SLEDAI and lipoproteins in the SLE subjects

We further assessed the relationship of lipoproteins with SLEDAI assessing the activity of SLE via univariate correlation analyses [16, 17]. Positive correlations were observed between SLEDAI and TG (*r* = 0.30; *P* < 0.01), and VLDL-C (*r* = 0.26; *P* < 0.01) (Table 3). Simultaneously, univariate correlation analyses also revealed negative correlations between SLEDAI and HDL-C (*r* = − 0.28; *P* < 0.01), LDL-C (*r* = − 0.18; *P* = 0.02), Apo A (*r* = − 0.31; *P* < 0.01), and Apo B (*r* = − 0.23; *P* < 0.01).

**Table 3:** Clinical correlation between SLEDAI and lipoproteins in the SLE subjects (*n* = 71)

Parameter	SLEDAI	
	*r*	*P*-value
Total cholesterol (TC, mmol/L)	−0.13	0.09
Triglyceride (TG, mmol/L)	0.30	< 0.01
HDL-C (mmol/L)	−0.28	< 0.01
LDL-C (mmol/L)	−0.18	0.02
VLDL-C (mmol/L)	0.26	< 0.01
Apolipoprotein A (Apo A, g/L)	−0.31	< 0.01
Apolipoprotein B (Apo B, g/L)	−0.23	< 0.01
Apolipoprotein E (Apo E, mg/L)	0.09	0.24

## Discussion

Recently, increasing studies have found that dyslipidemia in patients with SLE plays a key role in the development of atherosclerosis [7–10, 18]. It seems that atherosclerosis frequently appears during the process of SLE. Therefore, understanding the function of lipid metabolism in SLE is of great value and clinical benefits. In the present study, we assessed lipid profile and the relationship between dyslipidemia and disease activity of SLE in young female adults newly diagnosed with SLE. Our results showed that TG and VLDL levels were significantly increased, and HDL-C and Apo A were reduced in the subjects with SLE as compared to the control; there was positive correlations between SLE disease activity index and TG and VLDL-C.

Of note, in the present study, the SLE subjects compared to the control showed lower TC, LDL-C and Apo B levels. Previous clinical investigations have found that the young patients with SLE might also have a higher risk of cardiovascular disease, but the exact mechanisms by which SLE disease activity results in dyslipidemia remain to be addressed [19, 20]. It has been proved that high TG levels and low HDL-C levels resulted in elevated LDL oxidation, which was increased in SLE [21, 22]. Besides, because of chronic activation of the immune system in SLE, enhanced antibodies to LDL oxidation epitopes has been found in SLE, which could increase the accumulation of oxidized LDL to the arterial wall via Fc-receptor, leading to reduced LDL-C levels [22, 23]. Inconsistent with our results, recent study showed that TC levels were increased in the patients with SLE, and no significant difference in LDL-C was found between the SLE patients and the control [11]. Thus, more clinical investigations based on larger cohorts are warranted.

It has been shown that the patients with active SLE exhibited upregulation of TG and VLDL-C levels and downregulation of HDL-C levels, suggesting that dyslipidemia was aggravated in active SLE [22, 24], demonstrating aggravated dyslipidemia in the patients with inactive SLE [25]. In accordance with these results, our study by univariate correlation analyses revealed negative correlations between SLEDAI and HDL-C, LDL-C, Apo A, and Apo B; positive correlations between SLEDAI and TG, and VLDL-C, also indicating that SLE disease activity is strongly associated with the changes of lipid profile.

Because there are many factors affecting lipid metabolism, the selection of inclusion criteria could lead to the different conclusion. For example, the SLE patients with renal impairment showed higher TG and VLDL-C levels, and lower HDL-C levels; treatment with corticosteroids in the patients with SLE also showed growing dyslipidemia [26–28]. Thus, renal impairment and glucocorticoid treatment are involved in dyslipidemia in the patients with SLE. Additionally, diabetes mellitus, cardiovascular disease, liver and thyroid disease could also lead to changes in lipid profile [29–31]; menopaused or pregnant subjects showed increased TC and LDL-C [32]; abnormal BMI, smoking, alcohol use or taking any drugs known to influence lipid metabolism or the endocrine system could affect the results [33–35]. Therefore, based on these restricted inclusion criteria, our present analysis was able to assess the effects of the disease itself on lipid metabolism. However, further large population-based prospective studies are also necessary to explore the dyslipidemia in SLE patients.

The present study has potential clinical application by demonstrating dysregulated serum lipid profile and its correlation to disease activity in young female adults diagnosed with SLE. The SLE disease activity is strongly associated with the changes of lipid profile, with negative correlations between SLEDAI and HDL-C, LDL-C, Apo A, and Apo B and positive correlations between SLEDAI and TG, and VLDL-C. The lipid dysregulation metabolism could be potentially utilized in SLE diagnosed and severity evaluation.

## Conclusions

In summary, our results demonstrate that TG and VLDL levels are significantly increased, and TC, HDL-C, LDL-C, Apo A and Apo B levels are reduced in the young female SLE as compared to the control. Furthermore, univariate correlation analyses showed negative correlations between SLEDAI and HDL-C, LDL-C, Apo A, and Apo B; positive correlations between SLEDAI and TG, and VLDL-C. Therefore, SLE disease activity itself directly is associated with the cause of dyslipidemia, supporting the notion that the young patients with SLE suffering from dyslipidemia might also have a higher risk of cardiovascular disease.

## Data Availability

The datasets used and/or analysed during the current study are available from the corresponding author on reasonable request.

## Abbreviations

Apo A: Apolipoprotein A
Apo B: Apolipoprotein B
Apo E: Apolipoprotein E
BMI: Body mass index
C3: Complement 3
C4: Complement 4
CRP: C-reactive protein
ESR: Erythrocyte sedimentation rate
HDL-C: High-density lipoprotein-cholesterol
LDL-C: Low-density lipoprotein-cholesterol
SLE: Systemic lupus erythematosus
SLEDAI: SLE disease activity index
VLDL-C: Very low-density lipoprotein-cholesterol
